# Molecular characterization and combined genotype association study of bovine cluster of differentiation 14 gene with clinical mastitis in crossbred dairy cattle

**DOI:** 10.14202/vetworld.2016.680-684

**Published:** 2016-07-01

**Authors:** A. Sakthivel Selvan, I. D. Gupta, A. Verma, M. V. Chaudhari, A. Magotra

**Affiliations:** Molecular Genetics Laboratory, Dairy Cattle Breeding Division, National Dairy Research Institute, Karnal, Haryana, India

**Keywords:** cluster of differentiation 14, combined genotypes, *Helicobacter pylori 188I*, *Haemophilus influenzae I*, mastitis, single nucleotide polymorphisms

## Abstract

**Aim:**

The present study was undertaken with the objectives to characterize and to analyze combined genotypes of cluster of differentiation 14 (CD14) gene to explore its association with clinical mastitis in Karan Fries (KF) cows maintained in the National Dairy Research Institute herd, Karnal.

**Materials and Methods:**

Genomic DNA was extracted using blood of randomly selected 94 KF lactating cattle by phenol-chloroform method. After checking its quality and quantity, polymerase chain reaction (PCR) was carried out using six sets of reported gene-specific primers to amplify complete KF CD14 gene. The forward and reverse sequences for each PCR fragments were assembled to form complete sequence for the respective region of KF CD14 gene. The multiple sequence alignments of the edited sequence with the corresponding reference with reported *Bos taurus* sequence (EU148610.1) were performed with ClustalW software to identify single nucleotide polymorphisms (SNPs). Basic Local Alignment Search Tool analysis was performed to compare the sequence identity of KF CD14 gene with other species. The restriction fragment length polymorphism (RFLP) analysis was carried out in all KF cows using *Helicobacter pylori 188I* (*Hpy188I*) (contig 2) and *Haemophilus influenzae I* (*HinfI*) (contig 4) restriction enzyme (RE). Cows were assigned genotypes obtained by PCR-RFLP analysis, and association study was done using Chi-square (*χ*^2^) test. The genotypes of both contigs (loci) number 2 and 4 were combined with respect to each animal to construct combined genotype patterns.

**Results:**

Two types of sequences of KF were obtained: One with 2630 bp having one insertion at 616 nucleotide (nt) position and one deletion at 1117 nt position, and the another sequence was of 2629 bp having only one deletion at 615 nt position. ClustalW, multiple alignments of KF CD14 gene sequence with *B. taurus* cattle sequence (EU148610.1), revealed 24 nt changes (SNPs). Cows were also screened using PCR-RFLP with *Hpy188I* (contig 2) and *HinfI* (contig 4) RE, which revealed three genotypes each that differed significantly regarding mastitis incidence. The maximum possible combination of these two loci shown nine combined genotype patterns and it was observed only eight combined genotypes out of nine: AACC, AACD, AADD, ABCD, ABDD, BBCC, BBCD, and BBDD. The combined genotype ABCC was not observed in the studied population of KF cows. Out of 94 animals, AACD combined genotype animals (10.63%) were found to be not affected with mastitis, and ABDD combined genotyped animals was observed having the highest mastitis incidence of 15.96%.

**Conclusion:**

AACD typed cows were found to be least susceptible to mastitis incidence as compared to other combined genotypes.

## Introduction

In India, out of total milk production (132.4 MT), 45% milk (59.80 MT) is contributed by cattle, whereas out of total cattle milk production more than half (32.3 MT) is contributed by crossbred cattle [1]. The prevalence of bovine mastitis ranged from 29.34% to 78.54% in cows [2-4], and in the last five decades, economic losses due to mastitis have increased from 52.9 crores per annum in 1963 [5] to 7165.51 crores per annum in 2012 [6].

There is sizeable evidence that suggests resistance to non-infectious diseases such as bovine leukocyte adhesion deficiency [7], bovine chondrodysplastic dwarfism [8], and dermatophilosis [9] and infectious diseases such as tuberculosis, salmonellosis [10], Jhone’s disease [11], brucellosis [12], bovine leukemia virus infection [13], foot and mouth disease [14], and mastitis [15] have genetic basis. Therefore, present efforts are directed toward approaches such as identification of resistance genes, quantitative trait loci, and markers for such diseases so that disease can be detected at an early stage or a disease-resistant animal can be selected for further breeding [16]. There are a number of reports for candidate gene association with mastitis in *Bos* *taurus* [17-21] and also for *Bos* *indicus* animals [22,23].

Cluster of differentiation 14 (CD14) is a 55-k Dalton glycosyl phosphatidylinositol-anchored surface glycoprotein that is expressed mainly on monocytes and macrophages, and weakly on polymorphonuclear neutrophils surface [24]. It also acts as opsonin receptor, thereby helps in recognition and destruction of invading agents such as bacteria. Because of this role, as suggested by Ogorevc *et al*. [20,25], CD14 gene is one of the excellent candidates for mastitis in cattle. Ibeagha-Awemu *et al*. [19] and Pal *et al*. [26] characterized CD14 gene in Canadian Holstein and Jersey and Vrindavani Crossbred cattle breeds, respectively, while Kumar *et al*. [27] found an association between genetic variants of CD14 gene with mastitis incidence in Sahiwal (*B. indicus*) cattle. However, there is no report on CD14 gene characterization, single nucleotide polymorphisms (SNPs) detection, and combined genotype study in Karan Fries (KF) cattle (*B. indicus ×* *B. taurus*) which is one of the important crossbred cattle in India. Therefore, a study was planned to characterize and identify SNPs in complete CD14 gene and explore the possible association between genetic variants of CD14 gene in KF cattle (*B. indicus ×* *B. taurus*) with clinical mastitis using combined genotyping.

## Materials and Methods

### Ethical approval

The experiment was approved by Institutional Animal Ethics Committee.

### PCR-RFLP analysis

Blood samples were collected from randomly selected 94 KF cows maintained at cattle yard of National Dairy Research Institute, Karnal. Cows with history of incidences of clinical mastitis (affected ≥ once) and also non-affected cows were selected. Genomic DNA isolation was done by phenol-chloroform method as described by Sambrook and Russell [28] with minor modifications. Quality of genomic DNA was checked on 0.8% agarose gel electrophoresis. Quality and quantity of DNA were also estimated by nanodrop spectrophotometer method.

Six sets of forward and reverse gene-specific primers reported by Ibeagha-Awemu *et al*. [19] and Kumar *et al*. [27] were used to amplify complete KF CD14 gene. Primers were synthesized and procured from M/s. Eurofins Genomics India Pvt., Ltd., Bengaluru. The sequence of primers, their respective nucleotide numbers, target region, and amplicon sizes are given in Table-1.

**Table-1:** Sequence of the primers referred for amplification of complete CD14 gene in KF cattle.

Primer set	Sequence (5’-3’)		Number of base pairs	Target region	Amplicon size (bp)
1	F	ATTACCTTCTTCTGCACCTCCA	22	−404-307	711
	R	GAAAGTGAAGTCGCTCAGTCCT	22	(Promoter)	
2	F	ACACACCTGGAGAAGGCAA	20	177-729	553
	R	TCCAAGGGCTAGTTCCAG AG	20	(Promoter*)	
3	F	CAATTCCTGGTCAGGGAACTAA	22	561-1173	613
	R	GGCAGCCTCTGAGAGTTTATGT	22	(Promoter*)	
4	F	CTTCCTGTTATAGCCCCTTTCC	22	1012-1843	832
	R	CACGATACGTTACGGAGACTGA	22	(Promoter*, Exon1, Intron, Exon2*)	
5	F	GGGTACTCTCGTCTCAAGGAAC	22	1722-2546	825
	R	CTGAGCCAATTCATTCCTCTTC	22	(Exon2*)	
6	F	ACCTGACTCTGGACGGAAATC	21	2347-3093	747
	R	TAC AGGAGAGCAACCCTGAAA	21	(Exon2*)	

* Overlapping and partial. CD14=Cluster of differentiation 14, KF=Karan Fries

The polymerase chain reaction (PCR) mixture was incubated in thermal cycler initially at 94°C for 2 min followed by 34 cycles of 94°C for 30 s, 60°C (contig 1, 4, 5, 6) or 59°C (contig 2, 3) for 30 s, 72°C for 40 s, and a final extension of 72°C for 10 min. The amplified PCR products were checked on 2% agarose gel to ensure amplification of target region. Amplified PCR products from all sets of primers were custom sequenced from both ends (5’ and 3’ ends) by M/s. SciGenom Labs Pvt., Ltd. Nucleotide sequences were visualized and edited using BioEdit software. The forward and reverse sequences for each PCR fragments were assembled to form a complete sequence for the respective region of KF CD14 gene. The multiple sequence alignments of the edited sequence with corresponding reference with reported *B. taurus* sequence (EU148610.1) were performed with ClustalW software to identify SNPs. Basic Local Alignment Search Tool (BLAST) analysis was performed to compare the sequence identity of KF CD14 gene with other species.

The restriction fragment length polymorphism (RFLP) analysis was carried out in all KF cows using *Helicobacter pylori 188I* (*Hpy188I)* (contig 2) and *Haemophilus influenzae I* (*HinfI*) (contig 4) restriction enzyme (RE). PCR amplified CD14 gene products of each animal were digested with 0.4 µl each of *Hpy188I* and *HinfI* RE at 37°C for 16 h. Fragments of RE digestion were separated on 2.5% agarose gel and photographed using gel documentation system. Cows were grouped as mastitis affected and not affected and were assigned genotypes obtained by PCR-RFLP analysis.

### Statistical analysis

Chi-square (*χ*^2^) test was performed to test whether gene variants were independent. Further, restriction fragments were utilized to construct combined genotypes.

## Results and Discussion

**Figure-1:**
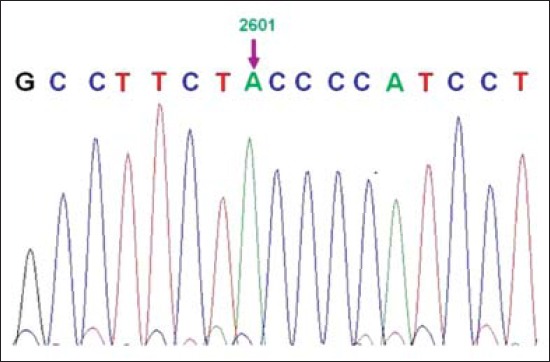
Chromatogram showing nucleotide change at position 2601 (G>A).

Two types of sequences of KF were obtained: One with 2630 bp having one insertion at 616 nucleotide (nt) position and one deletion at 1117 nt position, and the another sequence was of 2629 bp having only one deletion at 615 nt position. ClustalW, multiple alignments of KF CD14 gene sequence with *B. taurus* cattle sequence (EU148610.1) reported by Ibeagha-Awemu *et al*. [19], revealed 24 nt changes (SNPs) (Figure-1 and Table-2). Out of 24 SNPs compared with *B. taurus*, 13 SNPs were transition type of mutation, 8 SNPs were transversion type of mutation, 2 SNPs were deletion type of mutation, and 1 SNP was insertion type of mutation. 5 SNPs were in coding region resulting in synonyms amino acid change. Table-3 depicts comparative nucleotide sequences of Karan Fries (KF), *Bos taurus* and Sahiwal cattle showing nucleotide change of Guanine to adenine at position 2601. Crossbreeding and, species and breed variation of inheritance between KF (Tharparkar [*B. indicus*] × Holstein Friesian [*B. taurus*]), *B. taurus* may have been caused SNPs in KF cattle compared to reported CD14 gene sequence of *B. taurus* [19]. In BLAST analysis, KF cattle showed 86-99% sequence identity with several domestic animals (Table-4).

**Table-2:** Summary of nucleotide changes in CD14 gene of KF as compared to *B. taurus* (EU148610.1).

Region	Position	KF	*B. taurus* (Ibeagha *et al.*, 2008)
Promoter	269	A	G
	271	C	T
	273	A	C
	276	C	G
	277	A	G
	281	C	T
	357	T	G
	418	C	A
	431	A	G
	458	G	C
	615	Deletion	A
	616	A (insertion)	
	778	T	C
	922	A	G
	1117	Deletion	T
Exon 1	1239	G	T
	1291	C	T
Intron	1359	C	G
	1361	A	G
Exon 2	1811	A	G
	1909	G	A
	2276	T	C
Exon 2, 3’UTR	2601	A	G
	2621	G	T

*B. taurus*=*Bos taurus*, CD14=Cluster of differentiation 14, KF=Karan Fries

**Table-3:** Comparative nucleotide sequences of Karan Fries (KF), *Bos taurus* and Sahiwal cattle showing nucleotide change at position 2601 (G>A).

KF_2630	GCCTTCTACCCCATCCT	2610
KF_2629	GCCTTCTACCCCATCCT	2610
*Bos taurus*	GCCTTCTGCCCCATCCT	2610
Sahiwal	GCCTTCTGCCCCATCCT	2610

**Table-4:** Identity of CD14 gene of KF (*B.* *indicus×Bos taurus*) with other species.

Accession no.	Species	Homology (%)
EU148610.1	*B. taurus*	99
DQ457089.1	*B. bubalis*	98
NM_001077209.1	*O. aries*	97
DQ457090.1	*C. hircus*	93
AY753180.1	*S. scrofa*	86

*B. taurus*=*Bos taurus, B. bubalis*=*Bubalus bubalis, O*. *aries=Ovis aries, C. hircus=Capra hircus, S. scrofa=Sus scrofa,* CD14=Cluster of differentiation 14, KF=Karan Fries

PCR-RFLP analysis revealed two polymorphic patterns in contig 2 and 4. *Hpy188I* had cutting site in contig 2 that exhibited three genotypes (band patterns) such as AA (305 and 248 bp), AB (305, 248, 138 and 110 bp), and BB (305, 248, 138, and 110 bp) genotypes. *HinfI* had cutting site in contig 4 that exhibited three genotypes (band patterns) such as CC (377, 272, and 183 bp), CD (377, 272, 225, 183, and 47 bp), and DD (377, 225, 183, and 47 bp) genotypes. So, there were three genotypes in contig 2 (AA, AB, and BB) and three genotypes in contig 4 (CC, CD, and DD). Chi-square (*χ*^2^) analysis revealed that all the genotypes of KF cattle differed significantly from each other regarding mastitis incidence. The genotypes of both contigs (loci) number 2 and 4 were combined with respect to each animal to construct combined genotype patterns. If animal number “1” was having genotype “AA” at locus number 2, whereas genotype “CD” at locus number 4 then that animal will have combined genotype pattern “AACD.” In this way, combined genotypes were constructed. The maximum possible combination of these two loci shown nine combined genotype patterns, and it was observed only eight combined genotypes out of nine: AACC, AACD, AADD, ABCD, ABDD, BBCC, BBCD, and BBDD with 4, 20, 16, 10, 21, 3, 6, and 14 numbers of animals in each combined genotype, respectively (Table-5). The combined genotype ABCC was not observed in the studied population of KF cows.

**Table-5:** Frequency of combined genotype of CD14 gene in KF cattle.

Combined genotype	Total animals	Mastitis affected				Mastitis not affected			
	
		Number of animals	Percentage			Number of animals	Percentage		
	
			Within each genotype	Among mastitis affected (n=59)	Within herd (n=94)		Within each genotype	Among mastitis not affected (n=35)	Within herd (n=94)
AACC	04	02	50.0	03.39	02.13	02	50.0	05.71	02.13
AACD	20	10	50.0	16.94	10.63	10	50.0	28.57	10.63
AADD	16	8	50.0	13.55	08.51	8	50.0	22.85	08.51
ABCC	Not observed								
ABCD	10	9	90.0	15.25	09.57	1	10.0	02.85	01.06
ABDD	21	15	71.4	25.42	15.96	6	28.57	17.14	06.38
BBCC	03	1	33.3	01.69	01.06	2	66.66	05.71	02.12
BBCD	06	6	100.0	10.16	06.38	-	-		
BBDD	14	8	57.1	13.55	08.51	6	42.85	17.14	06.38
Total	94	59	-	100.00	62.77	35	-	100.00	37.23

CD14=Cluster of differentiation 14, KF=Karan Fries

### Association analysis of combined genotyped cows of CD14 gene with clinical mastitis

In Table-5, out of the four animals of combined genotypes “AACC,” 50% animals were mastitis affected and 50% animals were mastitis not affected. Among 59 animals (mastitis affected) and 94 animals (total animals analyzed for combined genotype study), 3.39% and 2.13% animals, respectively, were of “AACC” combined genotype. Similarly, among 35 animals (mastitis not affected) and 94 animals (total animals analyzed for combined genotype study), 5.71% and 2.13% animals, respectively, were of “AACC” combined genotype.

Among 94 cows studied for combined genotype analysis, 59 were mastitis affected. When the frequencies of combined genotypes of CD14 gene, fragments in KF cows were analyzed within each genotype: 100%, 90%, and 71.4% of animals in BBCD, ABCD, and ABDD genotype were observed having mastitis incidence, respectively. While among 59 mastitis affected KF cows, combined genotype ABDD was observed having the highest mastitis incidence (25.42%), i.e., 15 out of 59 animals. Out of 59 cows, around 64%, i.e., 38 cows with combined genotypes ABCD, ABDD, BBCD, and BBDD were mastitis affected which is in accordance with previous reports by Selvan *et al*. [29] where cows with AB and BB genotypes and by Selvan *et al*. [30] where cows with CD and DD genotypes were mastitis affected. Further, on comparison within herd (94 animals), combined genotype ABDD was observed having the highest mastitis incidence of 15.96%. Similar comparison was done for non-mastitis affected animals (35 animals). Out of 94 animals, AACD combined genotype animals (10.63%) were found to be not affected with mastitis (Table-5). Hence, it can be inferred that animals with ABDD genotypic combination are more susceptible to mastitis, and on the other hand, AACD typed animals are least susceptible to incidence of mastitis as compared to other combined genotypes.

## Conclusions

It may be possible that cows with AACD combined genotype might have a higher percentage of leukocytes expressing CD14 molecules on their surface which may increase the speed of response to pathogen attack [31,32]. Further research will be needed to conclude the relationships among CD14 genotypes, combined genotypes, concentration of CD14 in mammary tissues, and clinical mastitis.

## Authors’ Contributions

IDG conceived and designed the work. ASS conducted experiment. ASS and MVC done analysis, association study and AM assisted in writing of the manuscript. IDG and AV helped in revision of the manuscript. All authors read and approved the final manuscript.
